# Separation of Human P63^+^ Lung Progenitor Cells from Growth-Arrested Feeders Using Multidimensional Double Spiral Inertial Microfluidics for Efficient Bioprocessing

**DOI:** 10.1021/acs.analchem.5c08169

**Published:** 2026-05-13

**Authors:** Weilu Huang, Xuxia Zhu, Lanlan Zhu, Yang Qiao, Zheng Zhang, Kangkang Ren, Shaowen Li, Xinping Xu, Lu Yin

**Affiliations:** † Jiangxi Provincial Key Laboratory of Respiratory Diseases, Jiangxi Institute of Respiratory Disease, Department of Respiratory and Critical Care Medicine, The First Affiliated Hospital, Jiangxi Medical College, Nanchang University, Nanchang, Jiangxi 330209, China; ‡ Jiangxi Hospital of China-Japan Friendship Hospital, Nanchang, Jiangxi 330209, China; § Jiangxi Clinical Research Center for Respiratory Diseases, Nanchang, Jiangxi 330209, China

## Abstract

Recent clinical trials have highlighted the potential of P63^+^ lung progenitor cell (LPC) transplantation for lung repair and regeneration. Currently, *ex vivo* P63^+^ LPC expansion depends on coculture with growth-arrested fibroblast feeder cells (GAFs), necessitating repeated purification during passaging. While differential enzymatic digestion (DED) and fluorescence- or magnetic-activated cell sorting techniques (FACS/MACS) offer partial solutions, a scalable, efficient, and consistent separation technique remains unmet, particularly for cell therapy manufacturing. Here, we present a multidimensional double spiral (MDDS) inertial microfluidic device designed for high-throughput label-free enrichment of P63^+^ LPCs. The MDDS device achieves cell size-based separation of P63^+^ LPCs and growth-arrested feeder cells, processing at a rate of 10^6^ to 10^7^ cells per minute. MDDS-sorted P63^+^ LPC purity correlates with the LPC-to-GAF ratio in culture. With an initial ratio >1:1, it yields P63^+^ LPC purity exceeding 80%. Moreover, the device consistently recovers >80% of P63^+^ LPCs, with the unrecovered fraction enriched in senescent cells exhibiting compromised clonogenicity and differentiation capacity. In direct benchmarking against DED and FACS, the MDDS device delivered a balanced performance in terms of purity and recovery, while offering advantages in throughput, consistency, and scalability. We propose that this technology could enable more consistent and efficient enrichment of feeder-cultured P63^+^ LPCs, thereby supporting more robust clinical manufacturing processes.

## Graphical abstract

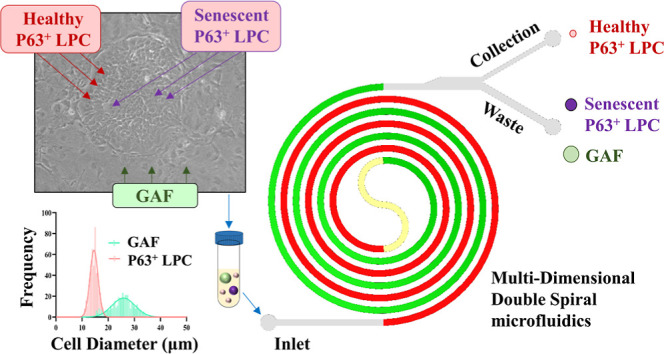

## Introduction

Respiratory diseases that cause lung damage represents a significant global threat to human health.^1^ Lung repair and regeneration rely on diverse populations of lung epithelial progenitor cells (LPCs).^2,3^ However, substantial loss of LPCs and genomic alterations within these cells have been documented in respiratory diseases,^4,5^ which may disrupt normal repair mechanisms and drive pathological structural remodeling. Transplantation of effective LPCs offers a promising therapy for respiratory disorders. Among various airway epithelial progenitor cells, P63^+^ basal progenitor cells (P63^+^ LPC) stand out as the leading candidate due to their critical role in regenerating both bronchial and alveolar tissues^6–8^ and the advancement of protocols for their isolation from patients and expansion at clinical scale.^9,10^ Recent pioneering clinical trials have provided preliminary evidence of the safety and efficacy of p63^+^ LPCs, underscoring their potential to treat a broad spectrum of respiratory diseases.^11–14^ Although protocols have been developed to generate lung epithelial lineages from pluripotent stem cells,^15,16^ these induced cells exhibit immature phenotypes, limiting their suitability for clinical application.

Culture expansion protocol is a critical determinant of the practicality and efficacy of P63^+^ LPC therapy. Current clinical methodologies for producing P63^+^ LPCs originate from the classical protocol established by Rheinwald and Green in the 1970s.^17,18^ Although remarkable improvements have been made in optimizing culture media and platforms for P63^+^ LPC expansion,^19,20^ the reliance on growth-arrested mouse embryonic fibroblasts (GAFs) as feeder cells, which is a cornerstone of the original protocol, remains essential for the stable long-term maintenance of the differentiation potential of these progenitor cells^11–14^ Consequently, this protocol requires the separation of P63^+^ LPCs from GAFs during passaging, followed by a terminal feeder-free passage to eliminate residual GAFs. In clinical trials, this separation has been achieved using differential enzymatic digestion (DED),^11–13^ whereas fluorescence- and magnetic-activated cell sorting (FACS and MACS, respectively) have been employed in laboratory investigations.^9,21^ However, these methods exhibit inherent limitations.

DED exploits the differential adhesiveness of P63^+^ LPC and GAFs to plastic surfaces during digestive enzyme-induced detachment.^22^ While this method is cost-effective and scalable for clinical-scale production, its consistency is compromised by variability in detachment timing across different cultures, posing challenges for standardized operating procedure. In contrast, FACS and MACS enable precise separation by targeting specific surface antigens, offering improved consistency. Nevertheless, their usages in clinical trials are hindered by suboptimal sorting efficiency, limited scalability, and high operational costs from equipment and reagents. The development of a technique that integrates the strengths of these approaches while mitigating their drawbacks holds significant value to advancing the clinical manufacturing of P63^+^ LPCs.

We previously employed a spiral inertial microfluidic device operating on the dean flow fractionation (DFF) principle to achieve high-throughput separation of cells exhibiting subtle size differences for cell therapy applications.^23,24^ This technology, initially derived from conventional DFF microfluidics with rectangular cross sections,^25^ was refined with a trapezoidal cross-section and later advanced into a multidimensional double spiral (MDDS) design to improve sorting efficiency and resolution.^26,27^ In this study, we customized the previously reported MDDS device to isolate P63^+^ LPC by exploiting size disparities between P63^+^ LPCs and GAFs. We show that the purity of MDDS-sorted P63^+^ LPC correlates with the LPC-to-GAF ratio in culture, achieving >80% purity at a 1:1 ratio. The recovery rate of P63^+^ LPC exceeds 80%, with the excluded progenitor cells exhibiting elevated senescence marker expression, reduced clonogenicity, and compromised differentiation potential *in vitro*. Furthermore, we compared the performance of the MDDS device with that of DED and FACS.

## Experimental Section

### Feeder Cell Preparation

The 3T3-J2 mouse embryonic fibroblast cell line (Shanghai Yaji Biotechnology, YS2148C) was cultured in high-glucose DMEM (Thermo Fisher Scientific, 11960044), supplemented with 10% BCS (Sigma-Aldrich, B7447), 1% penicillin–streptomycin (Thermo Fisher Scientific, 15140122), and 1% l-glutamine (Thermo Fisher Scientific, 25030081). Cells were maintained at 37 °C in a humidified incubator with 5% CO_2_. To induce growth arrest, 3T3-J2 cells were treated with 3 μg/mL mitomycin-C (Good Laboratory Practice Bioscience, GC12353) for 2 h at room temperature. Following thorough washing, growth-arrested cells were detached by trypsinization (Thermo Fisher Scientific, 25200056) and reseeded at a density of 5 × 10^4^ cells/cm^2^ to establish feeder layers for subsequent coculture with P63^+^ LPCs.

### Collection of Human Bronchial Tissue

Human bronchial tissue samples were collected under microscopic guidance from patients undergoing bronchoscopy examination at the First Affiliated Hospital of Nanchang University. Written informed consent was obtained from all patients in compliance with the ethical standards established by the hospital’s Medical Ethics Committee (approval number: (2023) CDYFYYLK (01-055)). The bronchoscopy-assisted sampling protocol was performed by board-certified respiratory physicians as previously described.^10^ Briefly, following standardized anesthesia administration, a sterile cytobrush (Changzhou Health Microport Medical Device, HM/102-20/1200Z) was inserted via a fiberoptic bronchoscope to gently collect bronchial tissues from bronchoscopically healthy regions of the third-to fourth-order bronchi.

### Isolation and Culture of P63^+^ LPC

Human bronchial tissues were subjected to enzymatic digestion in dissociation buffer containing 0.01% Trypsin–EDTA (Thermo Fisher Scientific, 25300062), 2 μg/mL protease XIV (Sigma-Aldrich, P5147), and 0.01 ng/mL DNase I (YEASEN, 89836) for 1 h at 37 °C under continuous gentle rocking. The resulting cell suspension was filtered through a 40 μm cell strainer (Biofil, CSS013040) to eliminate cell aggregates. After centrifugation and washing with 3:1 DMEM/F12 (Thermo Fisher Scientific, 11960044/11765054), the dissociated cells were seeded onto growth-arrested 3T3-J2 feeder layers. Cultures were maintained in a modified complete FAD medium (cFAD), as previously described,^7,8^ within a humidified incubator at 37 °C and 7.5% CO_2_. P63^+^ LPC colonies became detectable within 5 days. For long-term expansion, P63^+^ LPCs were routinely subcultured on feeder cells at a seeding density of 1 × 10^4^ cells/cm^2^. Alternatively, short-term feeder-free culture was achieved using collagen I-coated (Corning, 354249) vessels.

### Differential Enzymatic Digestion

To separate P63^+^ LPCs from cocultured GAFs, GAFs were selectively detached using 0.05% Trypsin–EDTA (Thermo Fisher Scientific, 25300062) for 3 min at 37 °C. This mild enzymatic treatment preferentially dissociated feeder cells while leaving P63^+^ LPC colonies intact. The residual adherent P63^+^ LPC colonies were subsequently harvested by incubation with 0.25% Trypsin–EDTA (Thermo Fisher Scientific, 25200056) for 5 min at 37 °C.

### FACS Analysis and Enrichment of P63^+^ LPCs

Feeder-free-cultured P63^+^ LPCs and GAFs were prelabeled with PKH26 (Solarbio, D0030) and CFSE (Thermo Fisher Scientific, C34554), respectively, and mixed at cell ratios of 1:10, 1:5, 1:1, and 2:1. Feeder-cultured P63^+^ LPCs were specifically labeled with a fluorophore-conjugated antihuman EpCAM antibody (BioLegend, 324206). The purity of P63^+^ LPCs separated via different separation methods was evaluated using a CytoFLEX S flow cytometry (Beckman Coulter Life Sciences) based on the fluorescent dye or antibody staining, with data analyzed using FlowJo software (BD Biosciences). For FACS-enrichment of feeder-cultured P63^+^ LPCs, a Cyto Expert SRT system (Beckman Coulter Life Sciences) was employed to positively select EpCAM^+^ cells under standard cell sorting configurations (2000 events/sec).

### MDDS Chip Design and Fabrication

The MDDS device architecture comprised two sequentially connected spiral microchannels with distinct cross-sectional geometries: a rectangular channel with 800 μm width and 100 μm height, and a trapezoidal channel with 800 μm width and inner and outer wall heights of 140 and 210 μm, respectively. The MDDS device was fabricated using polydimethylsiloxane (PDMS) via standard soft-lithography protocols as previously described.^27^ Briefly, fabrication began with the creation of a master mold, precision-milled from an alumina alloy substrate to match the specified channel dimensions (Shenzhen Kerch Technology, China). A PDMS prepolymer mixture (SYLGARD 184 Silicone Elastomer Kit, Dow Corning) was prepared in a 10:1 base-to-curing agent ratio, degassed under vacuum, cast onto the master mold, and thermally cured at 65 °C for 4 h. Fluidic access ports were created on the solidified PDMS chip after demolding. Finally, the PDMS chip was irreversibly bonded to a precleaned glass substrate using a COVANCE plasma machine (Femato Science).

### Cell Sorting Using MDDS Device

The MDDS device was primed with 1% Pluronic F-68 solution (Thermo Fisher Scientific, 24040032) for 1 min to prevent cell adhesion. The surfactant was thoroughly removed by flushing the channel with PBS. The cells to be sorted were suspended in cFAD medium containing 0.1% Pluronic F-68 at a concentration of 1 × 10^6^ to 3 × 10^6^ cells/mL. The cell suspension was loaded into a sterile syringe (BD, BD302995) and infused into the MDDS device through Tygon tubing using a R462 syringe pump (RWD Life Science) at a flow rate of 3–4 mL/min. Fluorescence-labeled cells sorted within the MDDS device were tracked using an EVOS M5000 imaging system (Thermo Fisher Scientific). Fluorescence images were processed using ImageJ software (NIH, USA). Unlabeled cell motion within the MDDS device was monitored in real-time using a MI52-N phase-contrast microscope (Mshot) equipped with an ×190 high-speed camera (Revealer) operating at 9300 fps to capture dynamic cell trajectories.

### Cell and Molecular Biology Assays

Detailed protocols for the following assays are provided in the Supporting Information: air–liquid interface culture, 3D Matrigel culture, immunofluorescence staining, quantitative real-time PCR, senescence-associated β-galactosidase staining and quantification, cell size measurement, population doubling time calculation, colony-forming assay, and cell cycle analysis.

### Statistics

Statistical significance was assessed using unpaired Student’s *t*-tests (P63^+^ LPCs vs GAF) and paired Student’s *t*-tests (collection vs waste; pre-vs postsorting; early vs late passage). One-way ANOVA with Tukey’s post hoc test was performed to compare the performance of MDDS across input samples with different LPC-to-GAF ratios, and the performance of various enrichment methods. A *P*-value < 0.05 was considered statistically significant.

## Results and Discussion

### Distinct Cell Size between P63^+^ LPCs and GAFs

Human P63^+^ LPCs were isolated from airway epithelial tissues collected via bronchoscopic brushing and cultured on GAFs to form colonies (Figure S1a). The colony identity was validated by immunofluorescence detection of the classical basal epithelial marker P63 and KRT5 (Figure S1b,c). The multipotent differentiation capacity of P63^+^ LPCs was demonstrated by their ability to generate stratified bronchial epithelium containing basal, ciliated, and secretory cell lineages under air–liquid interface (ALI) culture conditions (Figure S1d,e), as well as a minor population of hollow spherical organoids composed of alveolar lineages in Matrigel (Figure S1f–h). Significant morphological differences were observed between P63^+^ LPCs and GAFs. P63^+^ LPCs exhibited a characteristic typical cobblestone-like pattern, whereas GAFs showed a flattened spindle shape, likely induced by growth inhibition (Figure 1a,b). Both cell types retained a spherical morphology in suspension, with average cell diameters of 15.3 ± 0.74 μm for multidonor P63^+^ LPCs and 24.4 ± 1.49 μm for GAFs across culture batches (Figure 1c–e). This marked disparity in cell size suggests the feasibility of size-based cell separation methods for enriching P63^+^ LPCs. Although GAFs displayed greater size heterogeneity than P63^+^ LPCs (Figure 1f), the consistent size difference between the two populations is maintained by the growth-arrested state of the feeders, which promotes substantial cell enlargement.

**Figure 1:**
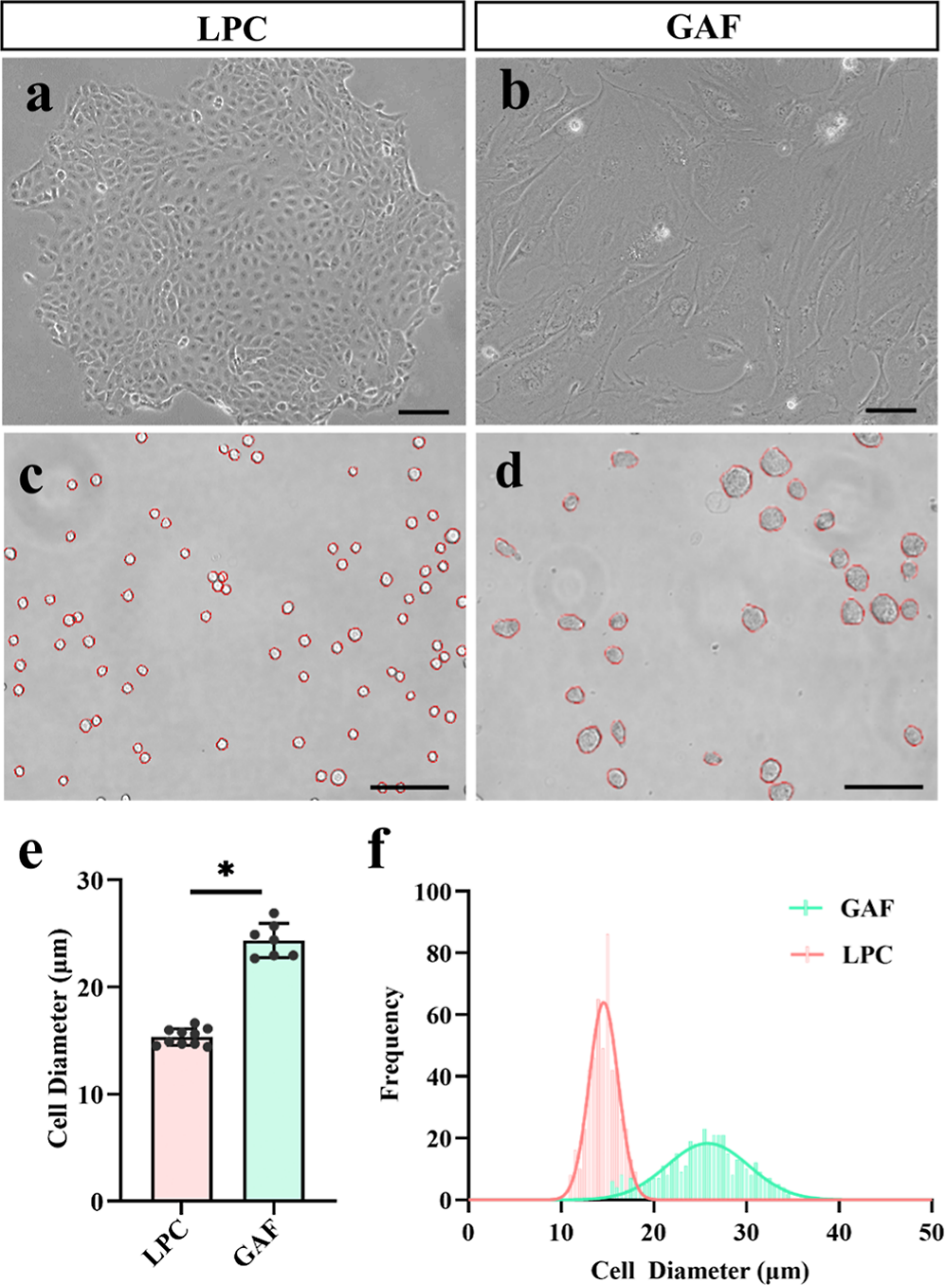
Cell size comparison of P63^+^ LPCs and GAFs. (a,b) Phase-contrast images of adherent P63^+^ LPCs (a) and GAFs (b). (c,d) Corresponding images of P63^+^ LPCs (c) and GAFs (d) in suspension. (e) Quantification of average cell diameter for P63^+^ LPCs (*n* = 10 donors) and GAFs (*n* = 7 independent cultures). (f) Cell size distribution histogram for both cell types from a representative culture. Scale bars: 100 μm. Error bars represent the standard deviation. * indicates *P* < 0.05 in unpaired Student’s *t*-test.

### Design and Optimization of MDDS Device for P63^+^ LPC Enrichment

An existing MDDS device design was adopted and tailored to separate P63^+^ LPCs from GAFs by size based on Dean flow fractionation principles.^26,27^ The device architecture comprises two spiral microchannels interconnected by a transitioning S-turn. The first spiral, with a rectangular cross-section, synchronizes the lateral positioning of all cells near the inner channel wall. The S-turn directs cells from the inner to outer wall before entry into the second spiral. The second spiral, featuring a trapezoidal cross-section, exploits cell size-dependent inertial forces to spatially separate cells into distinct lateral equilibrium positions (Figure 2a,b). A detailed description of the physical rational behind the MDDS design, along with the geometric customization for the specific cell sizes, is provided in the Supporting Information.

**Figure 2:**
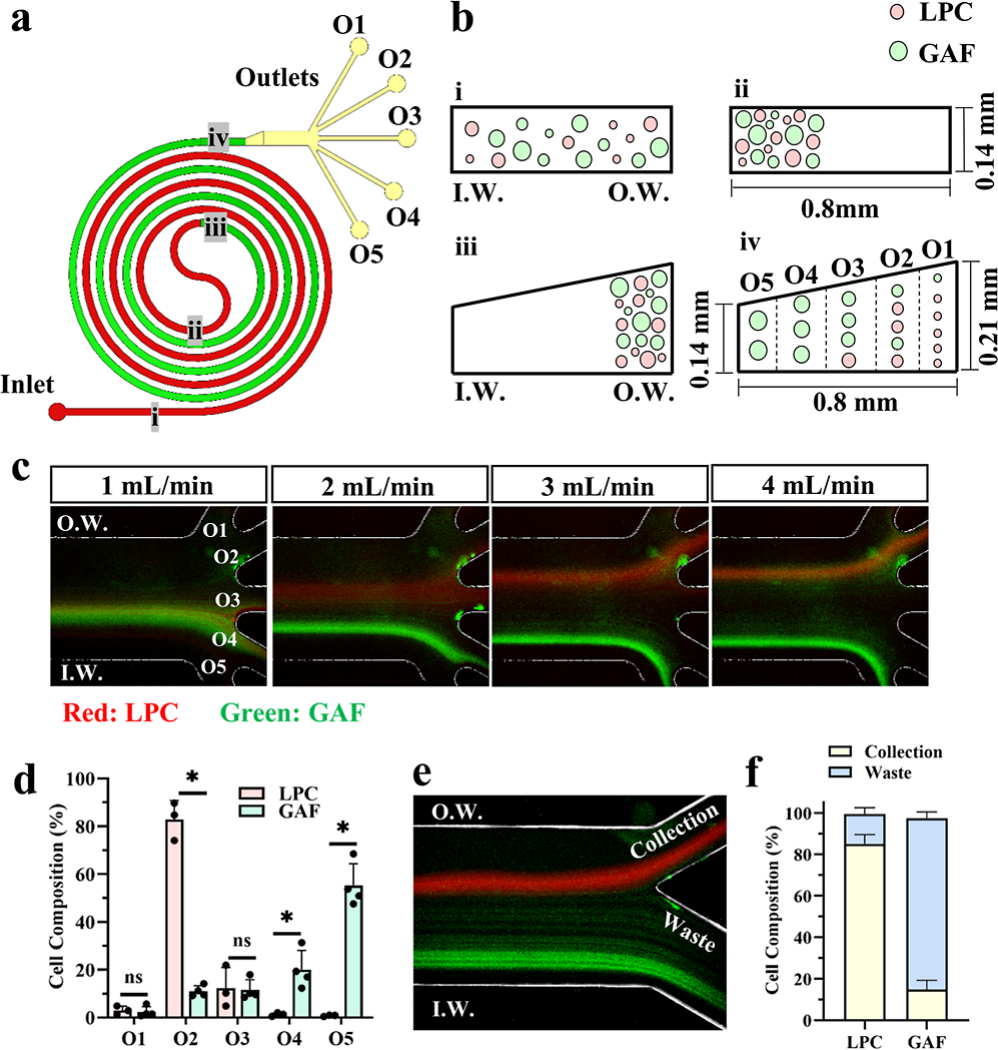
Design and functional validation of an MDDS device for P63^+^ LPC enrichment. (a,b) Schematic design of the 5-outlet MDDS microfluidic device. (b) Cross-sectional views at different parts of the MDDS channel, illustrating the separation mechanism: (i) mixed cell input, (ii) convergence at the inner wall after the first rectangular spiral, (iii) transition from inner to outer wall via the S-turn, and (iv) final differential inertial focusing in the second trapezoidal spiral based on cell size. (c) Fluorescence images showing the focusing positions of labeled P63^+^ LPCs and GAFs at different flow rates in the 5-outlet MDDS device. (d) Distribution profiles of P63^+^ LPCs and GAFs across the five outlets at 3.5 mL/min (*n* = 3 donors for P63^+^ LPCs; *n* = 4 for GAF cultures). (e,f) Validation of a dedicated 2-outlet MDDS device operated at 3.5 mL/min: (e) fluorescence images tracking cell focusing and (f) quantification of P63^+^ LPCs and GAFs sorted into the collection and waste outlets (*n* = 3 donors for P63^+^ LPCs; *n* = 3 for GAF cultures). Fluorescence images were acquired separately for LPCs and GAFs. Pseudo colors were assigned to each cell type, and the resulting overlays were processed using ImageJ software. All error bars represent the standard deviation. * indicates *P* < 0.05 in one-way ANOVA with Tukey’s post hoc test. ns indicates no statistical significance. I.W.: inner wall. O.W.: outer wall.

To optimize separation efficiency, a 5-outlet MDDS device was employed to test flow rates ranging from 1 to 4 mL/min. Fluorescence-labeled P63^+^ LPCs and GAFs were introduced, and their focusing behavior after the first spiral and before entry to the outlets was visualized. At flow rates of 2–4 mL/min, both cell types focused near the inner wall following the first spiral, indicating successful lateral position synchronization (Figure S2). Before entering the outlets, both populations formed broad focusing bands near the central line (outlets 3–4) at a flow rate of 1 mL/min (Figure 2c). Increasing the flow rate to 2 mL/min induced size-dependent migration: smaller P63^+^ LPCs shifted toward the outer wall, while larger GAFs focused closer to the inner wall (outlets 4–5). At 3 mL/min, P63^+^ LPCs stabilized near the outer wall (outlets 2–3), and further migrated toward outer wall (outlets 1–2) at 4 mL/min. In contrast, most GAFs remained near the inner wall at 3–4 mL/min, though a minor subpopulation overlapped with P63^+^ LPCs near the outer wall (outlet 2). Based on these dynamics, a flow rate of 3.5 mL/min was determined for subsequent separation experiments. Quantitative analysis at 3.5 mL/min confirmed distinct outlet distributions for P63^+^ LPCs and GAFs (Figure 2d), consistent with the fluorescence tracking (Figure 2c).

To streamline the separation of two cell types, the 5 outlets were merged to create a 2-outlet MDDS configuration. The collection outlet (outlets 1–2) was designated for P63^+^ LPCs, and the waste outlet (outlets 3–5) for GAFs. At 3.5 mL/min, the device achieved 85.2 ± 3.62% recovery of P63^+^ LPCs in the collection outlet and 82.6 ± 2.48% exclusion of GAFs into the waste outlet when the two populations were introduced separately (Figure 2e,f). Notably, ∼17.4% of GAFs contaminated the collection outlet, likely attributable to their inherent size heterogeneity.

The MDDS device inherently induces transient hydrodynamic stress on cells during sorting due to its rapid sorting speed. To assess the potential effects of this stress on P63^+^ LPCs, we evaluated essential functional properties of P63^+^ LPCs isolated from multiple donors pre- and postsorting. The results demonstrated that cell viability, proliferation rate, clonogenicity, and differentiation capacity were well-retained postsorting (Figure S3). These findings indicate that MDDS sorting imposes minimal impact on P63^+^ LPCs’ functionality, supporting its suitability as a clinical compatible cell sorting approach.

### Performance Evaluation of MDDS Device for P63^+^ LPC Enrichment

The MDDS device was further assessed for sorting mixed populations of P63^+^ LPCs and GAFs. To simulate coculture conditions reflecting progressive P63^+^ LPC expansion over feeder layer, differentially fluorophore-labeled P63^+^ LPCs and GAFs were mixed at ratios of 1:10, 1:5, 1:1 and 2:1. After sorting, cells from the collection and waste outlets were analyzed by FACS to assess the composition of differentially labeled cells. The purity of sorted P63^+^ LPCs in the collection outlet correlated positively with their prevalence in the input population, reaching 81.1% at a 1:1 ratio and 84.2% at a 2:1 ratio (Figure 3a,b). This suggests MDDS sorting achieves optimal performance in cocultures with dominant P63^+^ LPC population. Impurities in the sorted P63^+^ LPCs arose from a subset of GAFs exhibiting comparable cell size with the P63^+^ LPCs (Figure 1f), reflecting an intrinsic limitation of size-based MDDS sorting. Nevertheless, such moderate GAF contamination remains operationally acceptable during P63^+^ LPC passaging, as their growth-inhibited state prevents accumulation across passages, and would be eliminated during terminal feeder-free passage. Furthermore, recovery rates of P63^+^ LPCs remained >80.8% across all tested ratios (Figure 3c), underscoring the system’s robustness in retaining target cells irrespective of initial population composition.

**Figure 3:**
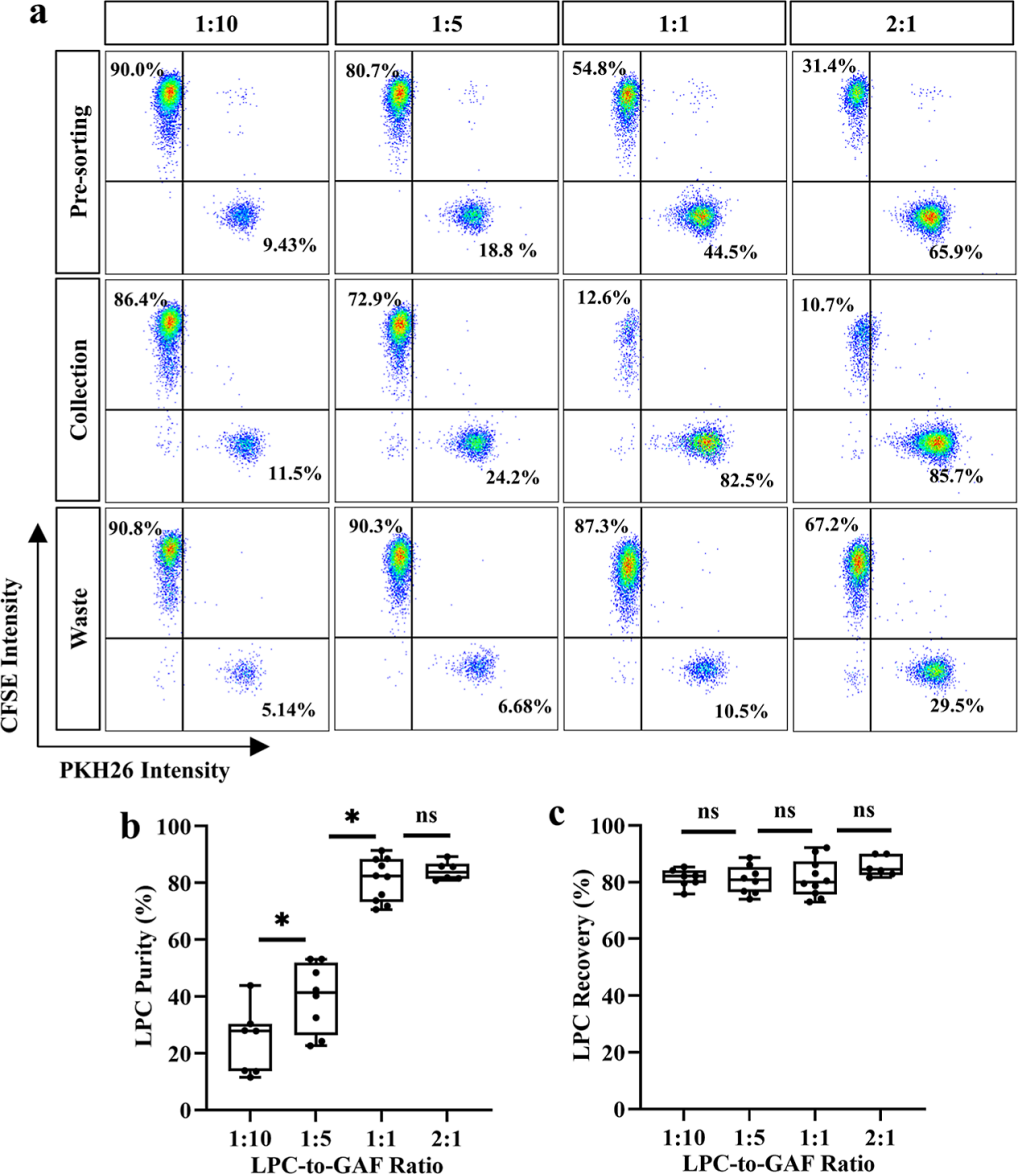
Enrichment of P63^+^ LPCs using MDDS device. PKH26-labeled P63^+^ LPCs and CFSE-labeled GAFs were mixed at specified ratios prior to sorting. (a) Representative FACS plots showing the cellular composition of presort mixtures and the corresponding postsort fractions from the device’s collection and waste outlets. (b,c) The purity (b) and recovery yield (c) of P63^+^ LPCs in the collection outlet, plotted against the initial P63^+^ LPC-to-GAF ratio. For LPC-to-GAF ratios of 1:10, 1:5, 1:1, and 2:1, P63^+^ LPCs from 7, 8, 10, and 3 donors were tested in 7, 8, 10, and 6 independent sorting experiments, respectively. * indicates *P* < 0.05, ns indicates no statistical significance in one-way ANOVA with Tukey’s post hoc test.

### MDDS Sorting Excludes Senescent and Functionally Compromised P63^+^ LPCs

The MDDS device actively excludes 15–20% P63^+^ LPCs into the waste outlet (Figure 3c). These excluded cells exhibit a markedly enlarged cell size compared to the smaller P63^+^ LPCs retained in the collection outlet (Figure 4a), a hallmark morphological feature of senescence.^28^ Consistent with this phenotype, the excluded population showed significantly elevated expression of senescence-associated genes, including p16, p21 and p53 (Figure 4b). Senescence-associated β-galactosidase assay further confirmed a significantly higher prevalence of senescent cells within the excluded fraction (Figure 4c). Functional assays demonstrated that the excluded P63^+^ LPCs display compromised proliferation (Figure 4d), clonogenicity (Figure 4e), organoid-forming capacity in Matrigel (Figures 4f and S4), and differentiation into bronchial epithelial lineages in ALI culture (Figure 4g and S4). Collectively, these data indicate that MDDS sorting reduces senescent cells and enriches for a functionally enhanced population of P63^+^ LPCs.

**Figure 4:**
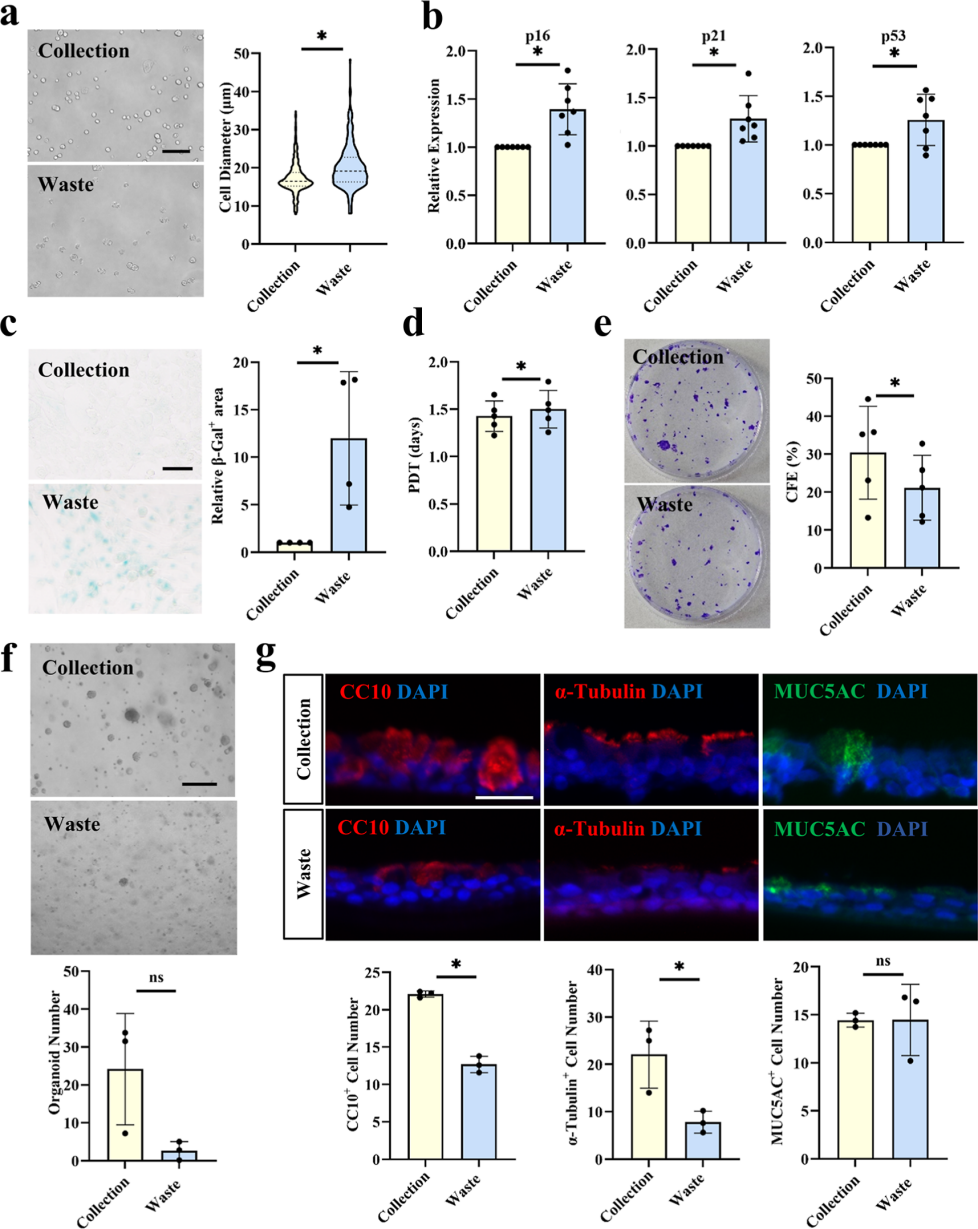
Phenotypic and function comparison of P63^+^ LPCs sorted into the collection and waste outlet. (a) Phase-contrast images (left) and quantification of cell diameter (right) for P63^+^ LPCs (*n* = 1 donor). (b) RT-qPCR analysis of senescence-associated gene expressions (*n* = 7 donors). (c) Bright-field images of senescence-associated β-galactosidase staining of P63^+^ LPCs (left) and quantification of the relative stained area (right, *n* = 4 donors). (d) Population doubling time (PDT, *n* = 5 donors). (e) Macroscopic images of crystal violet-stained P63^+^ LPC colonies (left) and the corresponding colony-forming efficiency (CFE, right, *n* = 4 donors). (f) Phase-contrast images of P63^+^ LPC-derived organoids in Matrigel (top) and quantification of organoid frequency per field of view (bottom, *n* = 3 donors). (g) Immunofluorescence images of P63^+^ LPC-derived bronchial epithelia stained for lineage markers: CC10 (club cell), acetylated α-tubulin (ciliated cell), and MUC5AC (goblet cell), with DAPI nuclei counterstain (top). The corresponding frequencies of each cell type per field of view are quantified (bottom, *n* = 3 donors). Scale bars: 100 μm (a,c); 400 μm (f); 30 μm (g). Error bars represent the standard deviation. * indicates *P* < 0.05, ns indicates no statistical significance in paired Student’s *t*-tests.

Cell age and cell cycle stage are known to influence the size of human stem cells, thereby might impact the MDDS sorting of P63^+^ LPCs.^29,30^ We found that the MDDS device excluded a significantly higher percentage of P63^+^ LPCs from late passages compared to early passages (19.61 ± 0.97% vs 9.10 ± 1.37%). This increased exclusion correlated with enlarged cell size and elevated senescence levels in later-passage cells (Figure S5a–d). However, cell cycle analysis revealed that the excluded P63^+^ LPCs contained a higher proportion of cells in the G2/M phase (22.00 ± 7.04% in waste vs 14.20 ± 6.43% in collection outlet), likely due to their transient size enlargement before mitosis (Figure S5e,f). This unintended loss of dividing cells was estimated to account for ∼3.26% of total P63^+^ LPC population.

### Comparison between MDDS Sorting and Conventional Enrichment Methods

The performance of the MDDS device was benchmarked against DED and FACS for enriching P63^+^ LPCs cultured on GAF feeder layer. The purity and recovery of P63^+^ LPCs were evaluated via FACS analysis of the percentage of EpCAM^+^ cells in the collected and discarded fractions obtained from each enrichment methods (Figure 5a). While FACS achieved superior sorting purity with minimal variability (95.48 ± 2.94%) compared to MDDS (82.41 ± 5.49%) and DED (71.76 ± 17.34%), both MDDS and DED exhibited significantly higher recovery rates (MDDS: 81.98 ± 1.88%; DED: 86.63 ± 14.86%; FACS: 57.76 ± 4.60%). MDDS provided greater purity with a comparable recovery yield relative to DED, while also demonstrating substantially less variation in both parameters (Figure 5b–c).

**Figure 5:**
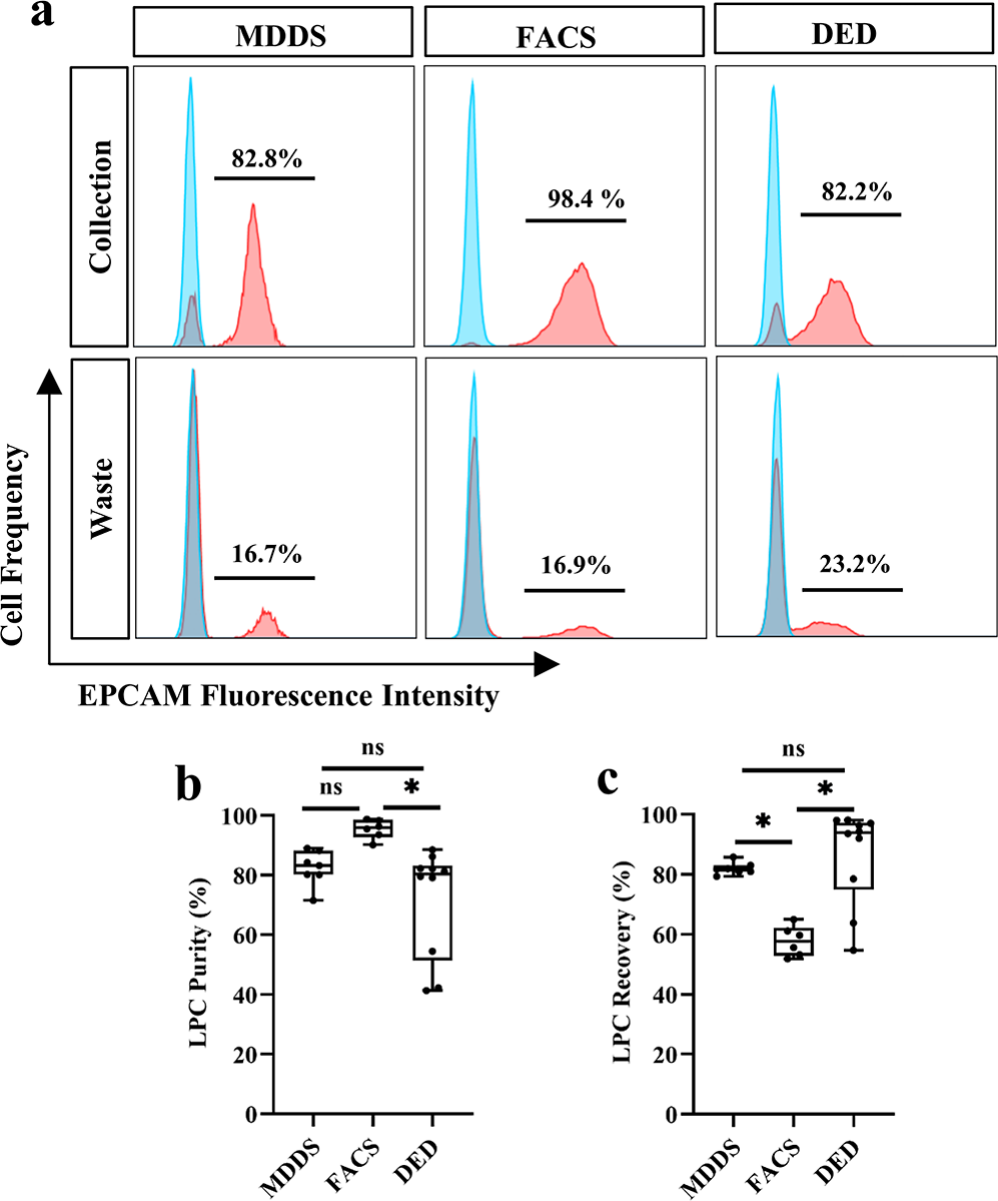
Comparison of MDDS, FACS and DED for enriching P63^+^ LPC. (a) Representative FACS analysis of cells enriched by each method, stained for human-EpCAM (red) versus an unstained control (blue). (b,c) The purity (b) and recovery (c) of P63^+^ LPCs achieved with each enrichment technique. For MDDS, FACS and DED, P63^+^ LPCs from 6, 4, and 9 donors were tested in 7, 6, and 10 independent enrichment experiments, respectively. * indicates *P* < 0.05, ns indicates no statistical significance in one-way ANOVA with Tukey’s post hoc test.

Notably, the MDDS sorting performance for feeder-cultured P63^+^ LPCs (Figure 5b,c) is comparable to that for feeder-free-cultured P63^+^ LPCs (Figure 3b,c), suggesting similar focusing behavior under both conditions. This interpretation is further supported by direct high-speed camera visualization of the focusing positions, comparing feeder-free-cultured and feeder-cultured, DED-enriched P63^+^ LPCs (Figure S6).

Taken together, these results highlight distinct trade-offs between the P63^+^ LPC enrichment methods. FACS, despite its unmatched purity and consistency, is constrained by suboptimal cell yield, high operational costs, and limited scalability, rendering it impractical for clinical-scale bioprocessing. DED, the current method for clinical P63^+^ LPC enrichment, offers scalability and cost-effectiveness with adequate purity and cell yield. However, its variability in both parameters necessitates operator expertise to achieve optimal efficiency. In contrast, the MDDS device strikes a balance, achieving higher cell recovery than FACS while surpassing DED in consistency. Notably, MDDS also retains critical advantages for clinical-scale processing, including high throughput, superior scalability, cost-effectiveness, and sufficient cell purity and yield. Furthermore, MDDS demonstrates superior capacity to reduce senescent and functionally compromised P63^+^ LPCs. A comprehensive comparison of the features, measured performance metrics, and qualitative assessments of these methods is summarized in Table 1.

**Table 1:** Comparison of P63^+^ LPC Enrichment Methods^a^

	**MDDS**	**FACS**	**DED**
**Working Principle**			
Separation Mechanism	hydrodynamic inertial focusing	specific surface antigen	plastic adherence
Cell-labeling	no	yes	no
**Measured Metrics**			
Purity	82.41 ± 5.49%	95.48 ± 2.94%	71.76 ± 17.34%
Recovery	81.98 ± 1.88%	57.76 ± 4.60%	86.63 ± 14.86%
**Qualitative Interpretation**			
Throughput	10^6^ to 10^7^ cells/min	10^5^ cells/min	N.A^b^
Cost	low	high	low
Scalability	good	poor	good
Exclusion of Senescent Cells	yes	yes^c^	no

a The purity and recovery metrics are derived from the experimental data shown in Figure 5. The throughput estimate for MDDS is based on the input sample processing rate, and that for FACS is based on the instrument set point; these operational parameters are detailed in the Experimental Section.

b Throughput is not applicable for DED due to the absence of a sorting step.

c Exclusion of senescent cells by FACS, although not validated in the present study, is feasible using surface markers of senescence

### Other Potential Applications and Limitations

DFF microfluidics has been employed across a broad spectrum of biomedical applications, most notably for the enrichment of rare cells from liquid biopsies.^25,31^ We propose that its high-throughput and label-free attributes offers a valuable strategy for selecting specific cell subpopulations in clinical-scale therapeutic cell manufacturing, an area with few alternatives. Accordingly, we have previously applied this technology to enrich functional subsets of chondrocytes and mesenchymal stromal cells from different tissues.^23,24,32^ Here, we further demonstrate its utility by integrating a tailored MDDS sorting into P63^+^ LPC bioprocessing. The applicability of this MDDS device may extend to other contact-dependent coculture systems, such as induced-pluripotent stem cells grown on mouse embryonic fibroblasts,^33^ where the mitotically inactivated feeder cells exhibit similar cell size enlargement.

The presented MDDS technology has several limitations, which we discuss here as important avenues for future improvements. First, while MDDS improves the consistency and purity of P63^+^ LPC processing over DED, it does not fully eliminate murine GAFs, which is an essential safety requirement for therapeutic products. Consequently, a terminal feeder-free passage, mirroring current clinical protocols,^11–13^ remains necessary to remove residual GAFs. Second, MDDS performance is highly dependent on the LPC-to-GAF ratio (Figure 3). Although a coculture at the time of passaging typically exhibits a ratio >1:1, which yields >80% purity, enhanced microfluidic resolution would extend the applicability of MDDS to the purification of rare stem cell populations from cocultures. Third, the size-based exclusion of senescent P63^+^ LPCs unintendedly depletes a minor subset of healthy cells undergoing transient, cell cycle-dependent enlargement (Figure S5). Despite this minimal loss, enriching a functional P63^+^ LPC population remains paramount for clinical manufacturing.

## Conclusion

We employed an MDDS inertial microfluidic device design and customized it to enrich feeder-cultured P63^+^ LPCs, addressing scalability and consistency challenges in clinical manufacturing. MDDS sorting achieves performance balance between conventional DED and FACS enrichment methods, combining high throughput, superior scalability, cost-effectiveness with favorable purity and yield. Notably, MDDS device uniquely enables the exclusion of aging cells with compromised functional potencies, demonstrating a critical advantage over conventional methods. The MDDS sorting process integrates seamlessly into existing P63^+^ LPC expansion protocols, presenting a viable alternative enrichment method for clinical-scale production.

## Supplementary Material



## Notes

It includes additional experimental settings and methods, supplementary experimental figures, and a detailed explanation of the working principle and customization of the MDDS device (PDF)
