# Supplementary material for: Separation of Human P63+ Lung Progenitor Cells from Growth-Arrested Feeders Using Multidimensional Double Spiral Inertial Microfluidics for Efficient Bioprocessing
Source: Anal Chem. 2026 May 13;98(20):14832–40. doi: 10.1021/acs.analchem.5c08169 (PMC13218359; doi:10.1021/acs.analchem.5c08169)
Supplement: Supplementary file 1 [file ac5c08169_si_001.pdf]

Supporting Information

Separation of human P63$^+$ lung progenitor cells from growth-arrested feeders using multidimensional double spiral inertial microfluidics for efficient bioprocessing

Weilu Huang[a, #], Xuxia Zhu[b, #], Lanlan Zhu[a, #], Yang Qiao [a], Zheng Zhang [a], Kangkang Ren[a], Shaowen Li[a], Xinping Xu[a, b, c], Lu Yin[a, b, c]

[a] Jiangxi Provincial Key Laboratory of Respiratory Diseases, Jiangxi Institute of Respiratory Disease, Department of Respiratory and Critical Care Medicine, The First Affiliated Hospital, Jiangxi Medical College, Nanchang University, Nanchang, Jiangxi, China 330209.

[b] Jiangxi Hospital of China-Japan Friendship Hospital, Nanchang, Jiangxi, China 330209.

[c] Jiangxi Clinical Research Center for Respiratory Diseases, Nanchang, Jiangxi, China 330209.

[#] These authors contribute equally to this work.

Correspondence: Lu Yin, Jiangxi Institute of Respiratory Disease, The First Affiliated Hospital of Nanchang University, 1519 Dong Yue Avenue, Nanchang, Jiangxi, China 330209. Email: yinlu@ncu.edu.cn.

Table of Contents

Supplementary Materials and Methods......S3

Supplementary Figures and Tables......S7

Supplementary Expanation: Working principle and customization of the MDDS device......S15

Supplementary Materials and Methods

Air-liquid interface (ALI) culture

$P63^+$ LPCs (2×10^5 cells) were seeded onto the apical chamber surface of 24-well Transwell® inserts (Corning, 3413) and maintained in cFAD medium (in both apical and basolateral chambers) at 37˚C with 7.5% CO_2 until a confluent monolayer formed in 24-48 hours. To initiate differentiation, the apical chamber medium was aspirated, and that in the basolateral chamber was replaced with PneumaCult-ALI Medium (STEMCELL Technologies, 05001). Cultures were maintained under such ALI conditions for 21 days with basolateral medium replenished every 2-3 days.

3D Matrigel culture

$P63^+$ LPCs (8×10^5 cells) were resuspended in 200 μL growth factor-reduced Matrigel® (BioGeous, M315066) and seeded in 24-well plates. The cell-laden Matrigel was polymerized in a humidified incubator at 37 °C with 7.5% CO_2 for 20 minutes. After solidification, airway organoid culture medium (BioGeous, K2018-HA) was gently added into each well. The organoids were cultured for 21 days, with medium replenished every alternative day.

Immunofluorescence staining

$P63^+$ LPC-derived bronchial epithelium and alveolar organoids were fixed with natural buffered formalin (Solarbio, G2161). For the ALI cultures, Transwell® membrane carrying differentiated bronchial epithelium was carefully excised and processed

through standard tissue processing, followed by paraffin embedding. For organoids cultured in Matrigel®, the organoid-containing Matrigel® was gently collected by scraping and embedded in 3% agarose gel, followed by standard tissue processing and paraffin embedding. Five-micron tissue sections were cut and mounted on poly-lysine-coated slides for subsequent staining. Standard immunofluorescence staining protocol was utilized to stain the tissue sections for antibodies against P63 (abcam, ab735), KRT5 (abcam, ab52635), pro-SPC (abcam, ab211326), PDPN (abcam, ab10288), MUC5AC (abcam, ab198294), CC10 (Santa Cruz, sc-365992), and α-tubulin (abcam, ab24610). Immunofluorescence images were acquired using a DMi8 fluorescence microscope (Leica).

Quantitative real-time PCR

Total RNA was extracted from $P63^+$ LPCs using TransZol Up RNA Extration Kit (TransGen Biotech, ET111-01-V2), and reverse transcribed into cDNA using Hifair® AdvanceFast One-step RT-gDNA Digestion SuperMix (Yeasen Biotechnology, 11151ES60). Quantitative real-time PCR was subsequently performed with Hieff UNICON™ Universal Blue qPCR SYBR Green Master Mix (Yeasen Biotechnology, 11184ES08) on a Quant Studio Dx PCR system (Thermo Fisher Scientific). The relative gene expression levels of p16, p21 and p53 were normalized to GADPH and quantified using the $2^{-\Delta\Delta C_t}$ method. The primers for human p16, p21, p53 and GAPDH are listed in Supporting Information Table S1.

Senescence-associated β-galactosidase staining and quantification

$P63^+$ LPCs (8×10^4 cells) sorted into the collection or waste outlet of the MDDS device were seeded in 6-well plates and allowed to adhere overnight. Cellular senescence was assessed using the Senescence β-Galactosidase Staining Kit (Beyotime Biotechnology, C0602) according to the manufacturer's protocol. Bright-field images of β-galactosidase staining were captured. The β-galactosidase-stained area per image was computed using an automated image analysis algorithm as we previously described [1]. For comparative analysis, the β-galactosidase-stained area of $P63^+$ LPCs from the waste outlet was normalized to that from the collection outlet.

Cell size measurement

Cell suspension (20 μL) was load into a Countstar® cell counting cassette (Alit Biotech, CO010101) for phase-contrast microscopy using an EVOS M5000 cell imaging system (Thermo Fisher Scientific). Cell diameter was quantified using a self-developed automated algorithm, which employs edge detection and erosion-based cell segmentation to delineate individual cell boundaries for precise morphometric analysis.

Population doubling time

$P63^+$ LPCs were expanded in collagen I-coated 6-well plates for 4-5 days at an initial seeding density of 2000 cells/cm^2. The cells were harvested and viable cells were counted using an NC-3000 NucleoCounter system (ChemoMetec). Population doubling time (PDT) was calculated according to the formula: $PDT = D / \log_2 (N_f / N_i)$, where D is the culture duration (days), N_i is the seeded cell count, and N_f is the harvested cell count.

Colony-forming efficiency

$P63^+$ LPCs (500 cells) were seeded in a collagen I-coated 60-mm petri dish and cultured for 10 days to allow colony formation. Colonies were fixed with 4% formaldehyde for 30 minutes and subsequently stained with 0.1% crystal violet solution (Beyotime, C0121) for 20 minutes at room temperature. Stained colonies were manual counted. Colony-forming efficiency (CFE) was calculated as: CFE = N / 500 × 100%, where N is the number of colonies counted per dish.

Cell cycle analysis

The cell cycle of $P63^+$ LPCs was analyzed by quantifying DNA content via DAPI staining using a NucleoCounter system (ChemoMetec, NC-3000), following the manufacturer's built-in protocol titled "Two-step cell cycle analysis".

Supplementary Figures and Tables

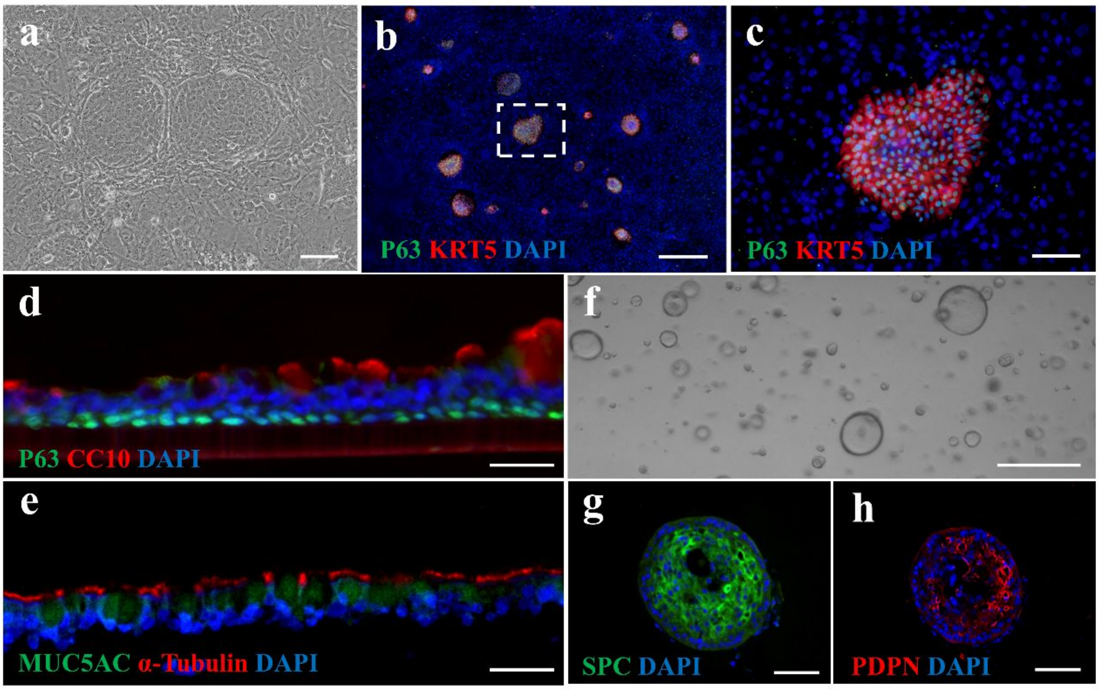

Figure S1. Clonal expansion and differentiation of P63⁺ LPCs. (a) Phase-contrast image of P63⁺ LPC clones expanding on GAF layer. (b-c) Immunophenotypic validation of LPC clones via co-immunofluorescence staining for P63 (green) and KRT5 (red). The boxed area in shown at higher magnification in (c). (d-e) Immunofluorescence analysis of p63⁺ LPCs following 14-day differentiation in ALI culture, demonstrating commitment to bronchial epithelial lineages. Co-staining for (d) p63 (green) and CC10 (club cell marker, red) and (e) MUC5AC (goblet cell marker, green) and acetylated α-tubulin (ciliated cell marker, red). (f) Bright-field image of organoids derived from P63⁺ LPCs in Matrigel at day 30. (g-h) Immunofluorescence analysis of P63⁺ LPC-derived organoids for alveolar lineage markers. (g) pro-SPC (alveolar type II cell marker, green) staining. (h) PDPN (alveolar type II cell marker, red) staining. Nuclei are counterstained with DAPI (blue) in (b), (c), (d), (e), (g), and (h). Scale bars: 100 μm (a, c, d, e, g, h); 500 μm (b, f); 30 μm (d, e).

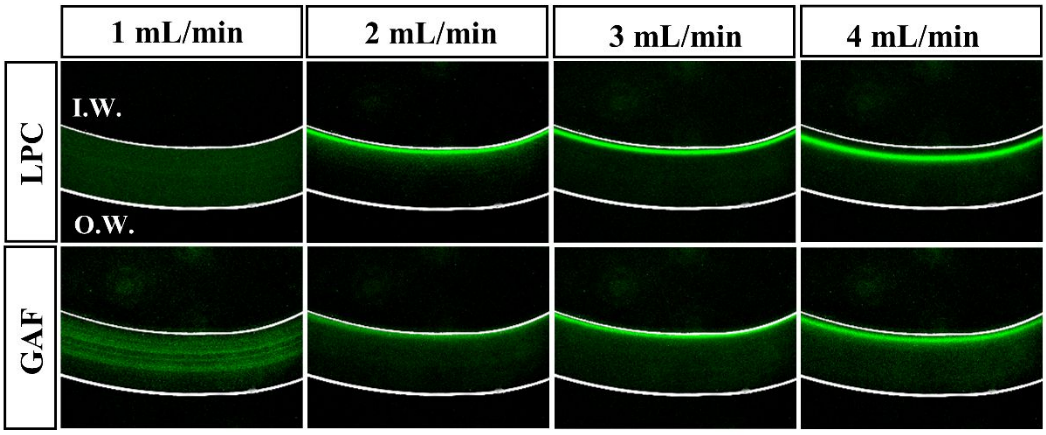

Figure S2. Synchronization of lateral cell positions via the first spiral channel of the MDDS device. Fluorescence images showing the focusing behavior of labelled P63$^+$ LPCs and GAFs at the end of the first spiral channel in the 5-outlet MDDS device, acquired at the indicated flow rates. I.W.: inner wall. O.W.: outer wall.

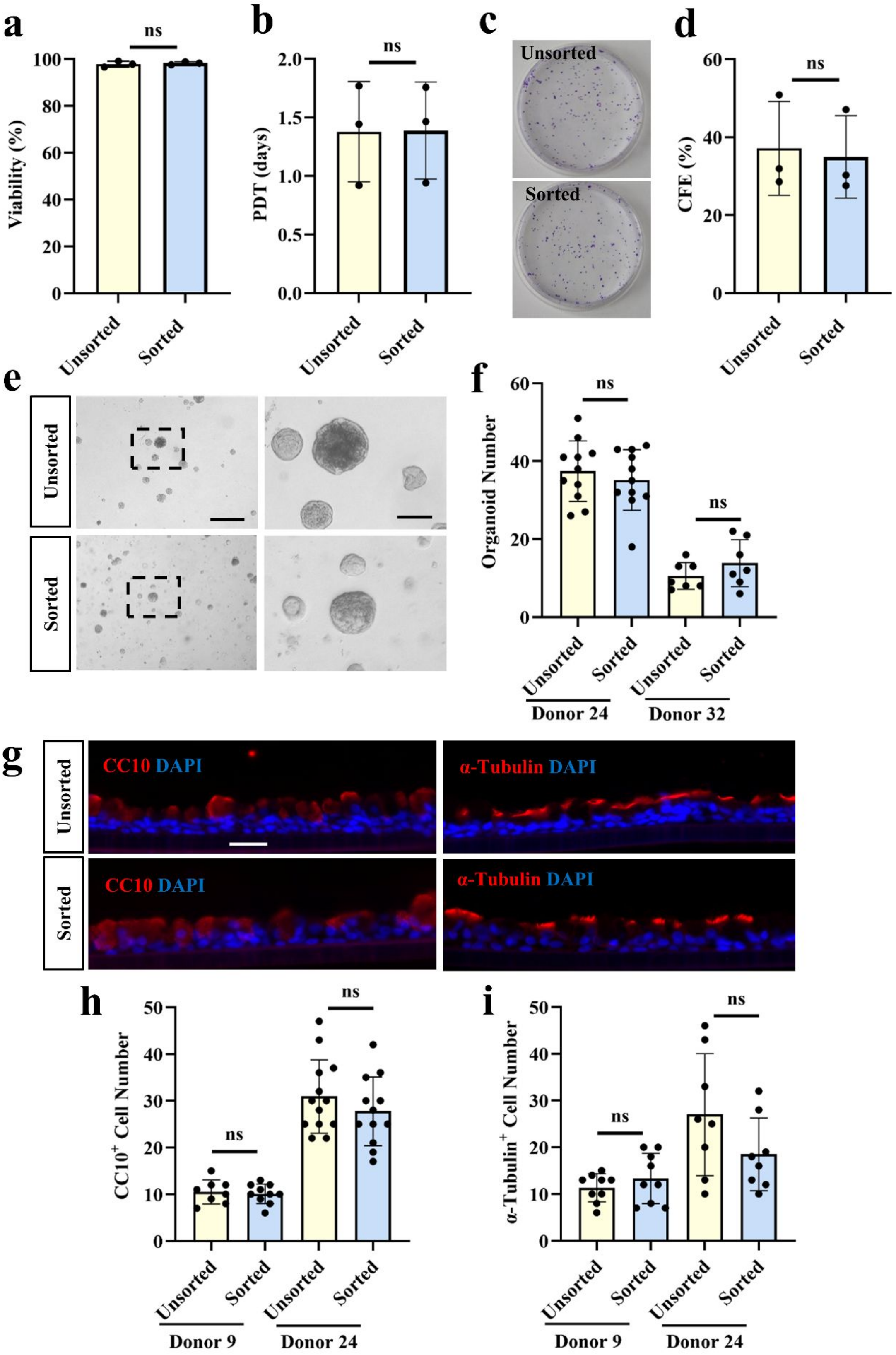

Figure S3. Functional characterization of P63⁺ LPC pre- and post-MDDS sorting. (a) Cell viability (n = 3 donors). (b) Population doubling time (PDT, n = 3 donors). (c-d) Colony-forming efficiency (CFE, n = 3 donors): (c) Macroscopic colony images

(crystal violet stain) and (d) quantified CFE. (e-f) Organoid-forming potential: (e) bright-field images of Matrigel-embedded organoids (boxed areas magnified at right) and (f) quantified organoid frequency per field of view. (g-i) Differentiation potential under ALI conditions: (g) immunofluorescence images of $P63^{+}$ LPC-derived bronchial epithelia stained for CC10 (club cell marker) and acetylated α-tubulin (ciliated cell marker), respectively, with DAPI nuclei counterstain; quantification of the (h) club cell and (i) ciliated cell frequency per field of view. Scale bars: 400 μm (e, left), 100 μm (e, right), and 30 μm (g). All error bars represent standard deviation. ns indicates no statistical significance ($P > 0.05$) in both paired (a b, and d) and unpaired (f, h, and i) Student's *t*-tests.

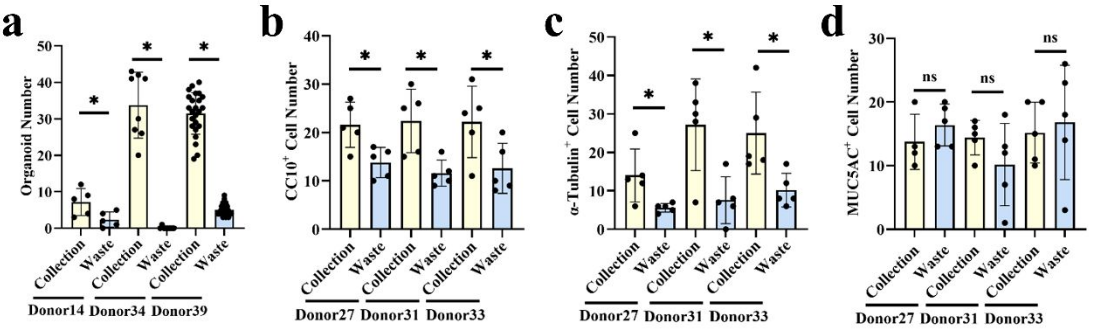

Figure S4. Comparison of the organoid-forming and differentiation capacity of multi-donor $P63^+$ LPCs sorted into the collection versus waste outlet. (a) Image-based quantification of the organoid frequency for 3 donors (organoid number per field of view). (b-d) Image-based quantification of the $CC10^+$ club cell (b), α-$tubulin^+$ ciliated cell, and $MUC5AC^+$ goblet cell frequencies for 3 donors (number of cells per field of view). All error bars represent the standard deviation. * indicates $P < 0.05$, ns indicates no statistical significance in unpaired Student's t-tests.

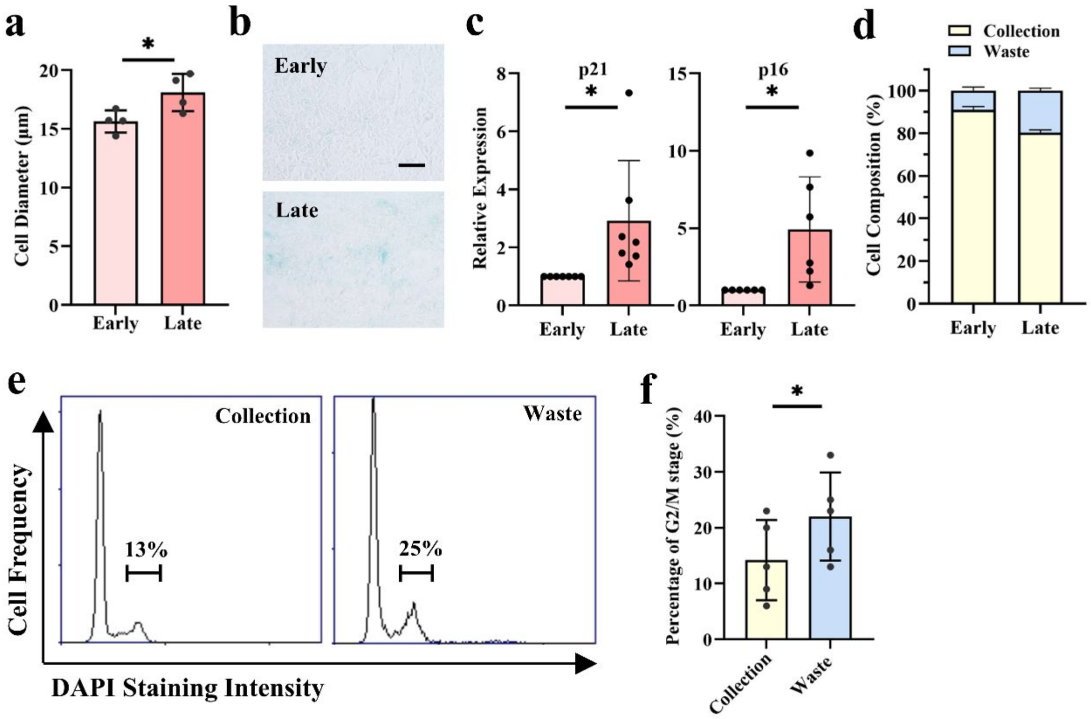

Figure S5. Effects of cell age and cell cycle stage on MDDS sorting of P63⁺ LPCs. (a) Cell diameter of P63⁺ LPCs at early (passage 1-3) and late (passage 4-6) passage (n = 4 donors). (b) β-galactosidase staining of P63⁺ LPCs at early and late passage. (c) RT-qPCR analysis of senescence-associated gene expressions, showing p21 (n = 7 donors) and p16 (n = 6 donors) mRNA levels in early- and late-passage P63⁺ LPCs. (d) Percentage of P63⁺ LPCs sorted into collection and waste outlets at early and late passages (n = 3 donors). (e-f) Representative FACS plots for cell cycle analysis of P63⁺ LPCs sorted into the collection and waste outlets (n = 5 donors). (f) Quantification of P63⁺ LPCs at G2/M stage in the collection and waste outlets. Scale bar: 100 μm. All error bars represent standard deviation. * indicates statistical significance ($P < 0.05$) in paired Student's t-tests.

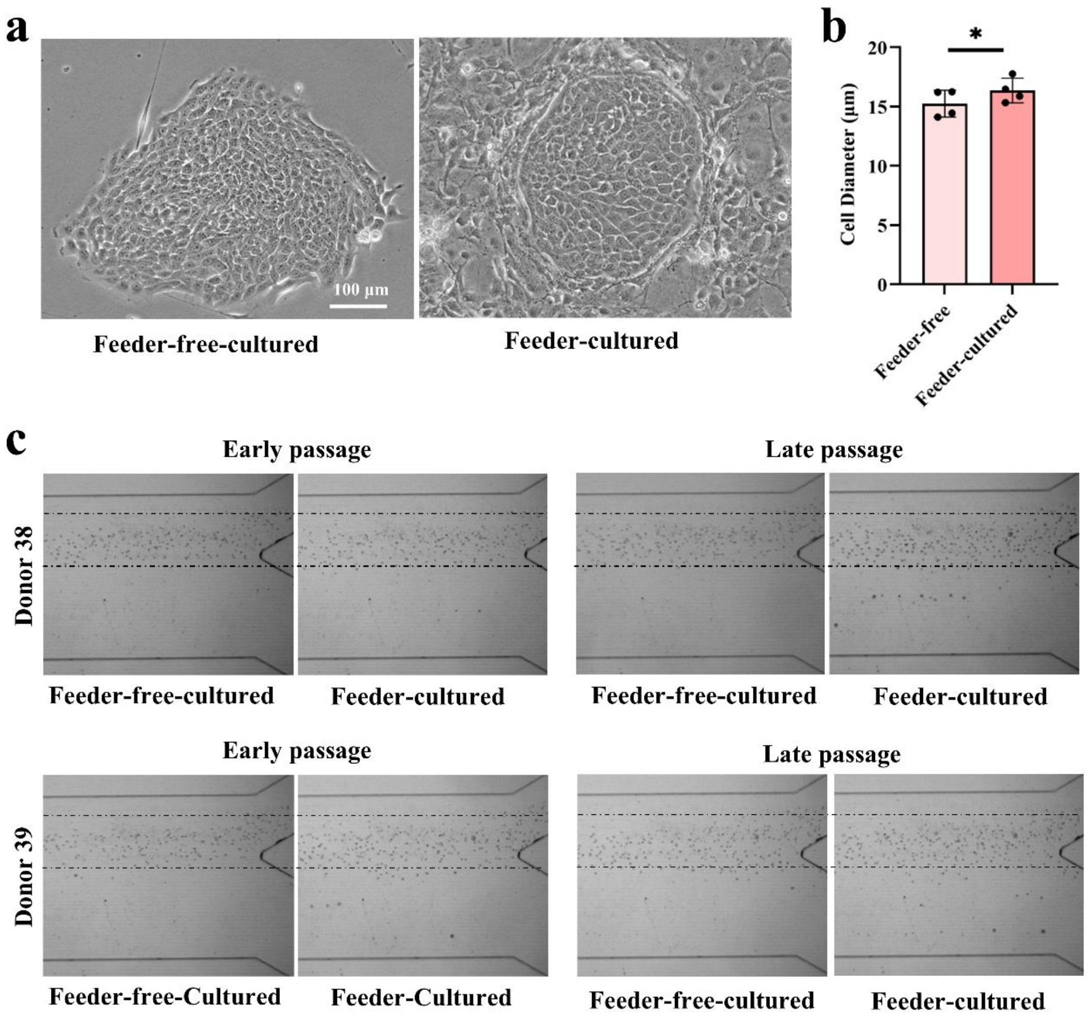

Figure S6. Comparison of the focusing behavior of feeder-free-cultured and feeder-cultured P63⁺ LPCs in the MDDS device. (a) Phase-contrast images of $P63^+$ LPCs cultured with or without GAFs. (b) Cell sizes of $P63^+$ LPCs cultured with or without GAFs. Data represents measurements from 2 donors at 2 passages. (c) High-speed camera images showing the focusing position of feeder-free-cultured and feeder-cultured, DED-enriched $P63^+$ LPCs at a flow rate of 3.5 mL/min in the MDDS device. Cells from 2 donors early (passage 1-3) and late (passage 4-6) passages were tested. Scale bar: 100 µm. All error bars represent standard deviation. * indicates statistical significance ($P < 0.05$) in paired Student's t-tests.

	Forward	Reverse
**p16**	GTGAGAGTGGCGGGGTC	CCCAACGCACCGAATAGTTA
**p21**	AGGTGGACCTGGAGACTCTCAG	TCCTCTTGGAGAAGATCAGCCG
**p53**	TGACACGCTTCCCTGGATTG	TCCGGGGACAGCATCAAATCA
**GAPDH**	CATACCAGGAAATGAGCTTG	ATGACATCAAGAAGGTGGTG

Table S1. List of human gene primers for RT-qPCR.

Supplementary Explanation: Working principle and customization of the MDDS device

As cells traverse a spiral microchannel, they are subjected to hydrodynamic forces in the transverse plane. These forces include: (i) a shear-induced lift force arising from the parabolic velocity profile of the flow, which drives cells away from the channel center; (ii) a wall-induced lift force resulting from the asymmetric wake generated as a cell approaches the channel wall; and (iii) a Dean drag force induced by centrifugal effects, which give rise to counter-rotating Dean vortices within the channel [2, 3]. The combined effect of these forces governs size-dependent cell migration and determines the equilibrium positions of cells in the transverse plane, thereby enabling the separation of cells based on size.

A key parameter that influences cell motion in the transverse plane is the confinement ratio ($CR = a/D_h$), defined as the ratio of cell diameter (a) to the hydraulic diameter of the microchannel ($D_h = 4A/P$, where A and P denote the area and perimeter of the channel's cross-section, respectively) [2]. In a spiral microchannel with a fixed D_h, small-size cells with a $CR < 0.01$ are predominantly influenced by the Dean drag force and become trapped within the Dean vortices. Conversely, large-size cells with a $CR > 0.07$ experience a strong net lift force sufficient to overcome the Dean drag force, causing them to focus into a stream near the inner channel wall. Cells of intermediate size, with CR values between these thresholds, exhibit partial focusing, characterized by a broad focusing band away from the inner wall.

Previous studies have introduced two critical advancements in the design of spiral microfluidic devices. The first involves the integration of an additional inlet for sheath flow [4], which initially positions all cells near the outer channel wall. This configuration synchronizes the transverse motion of small cells (CR < 0.01), enabling precise control over their Dean cycle and maximizing their separation from the focused stream of larger cells (CR > 0.07). It also facilitates the focusing of medium-size cells (0.01 ≤ CR ≤ 0.07), yielding a more sharply defined focusing band. The second advancement is the implementation of a trapezoidal cross-sectional geometry, in which the outer channel wall is deeper than the inner wall [5, 6]. This asymmetric design generates stronger Dean vortices in the outer side of the channel, resulting in a sharper transition between the outer "trapping" and inner "focusing" regimes in response to cell size and flow rate variations, thereby enhances the separation resolution.

The MDDS device incorporates and extends both design advancements [7, 8]. In our study, the first device compartment consists of a rectangular cross-sectional spiral engineered with a D_h of 177.8 μm (800 μm in width and 100 μm in height) to focus both $P63^+$ LPCs (CR = 0.086 > 0.07) and GAFs (CR = 0.137 > 0.07) near the inner channel wall. This is followed by an S-turn segment that reverses the spiral direction, thereby redirecting all cells to the outer wall. Together, these two components replicate the function of sheath flow used in previous two-inlet design [4], achieving inertial focusing-based cell synchronization without the throughput limitations associated with sample dilution by sheath fluid. The second spiral of the MDDS adopts a trapezoidal cross-sectional geometry to enable high-resolution separation. This customized section features a D_h

of 286.7 μm (800 μm in width, 140 μm and 210 μm in inner and outer heights, respectively), which facilitates the focusing of large GAFs (CR = 0.085 > 0.07) close to the inner wall, while retaining small P63$^+$ LPCs (CR = 0.053 < 0.07) within the outer partial focusing region.

Although the MDDS device was designed based on the Dean flow fractionation principle and tailored to the diameters of P63$^+$ LPCs (~24.4 μm) and GAFs (~15.3 μm), accurately predicting their transverse focusing positions and the optimal flow rate for achieving distinct separation remains challenging. To validate the device design, we first employed polystyrene beads (Rigorbiotech, PS02025/PS02015) of diameters comparable to the two cell types. The focusing behavior of 25 μm and 15 μm beads was experimentally characterized across flow rates ranging from 1 to 4 mL/min using a microscope (Mshot, MI52-N) equipped with a high-speed camera (Revealer, X190). The results confirmed successful focusing of both bead populations after the first rectangular spiral at flow rates of 3-4 mL/min, and clear separation after the second trapezoidal spiral at 2-3.5 mL/min (Supporting Information Figure S7).

The focusing behavior of P63$^+$ LPCs and GAFs within the MDDS device was subsequently visualized using fluorescence cell tracers under a fluorescence microscope (Thermo Fisher Scientific, EVOS M5000). Both cell types exhibited focusing patterns similar to those of the corresponding beads at the respective flow rates, although their precise focusing positions showed slight deviations (Figure 2 and Supporting Information Figure S2). These discrepancies are likely attributable to cell

deformability under high-speed flow conditions, which may result in a reduced effective cell size and thus influence hydrodynamic behavior.

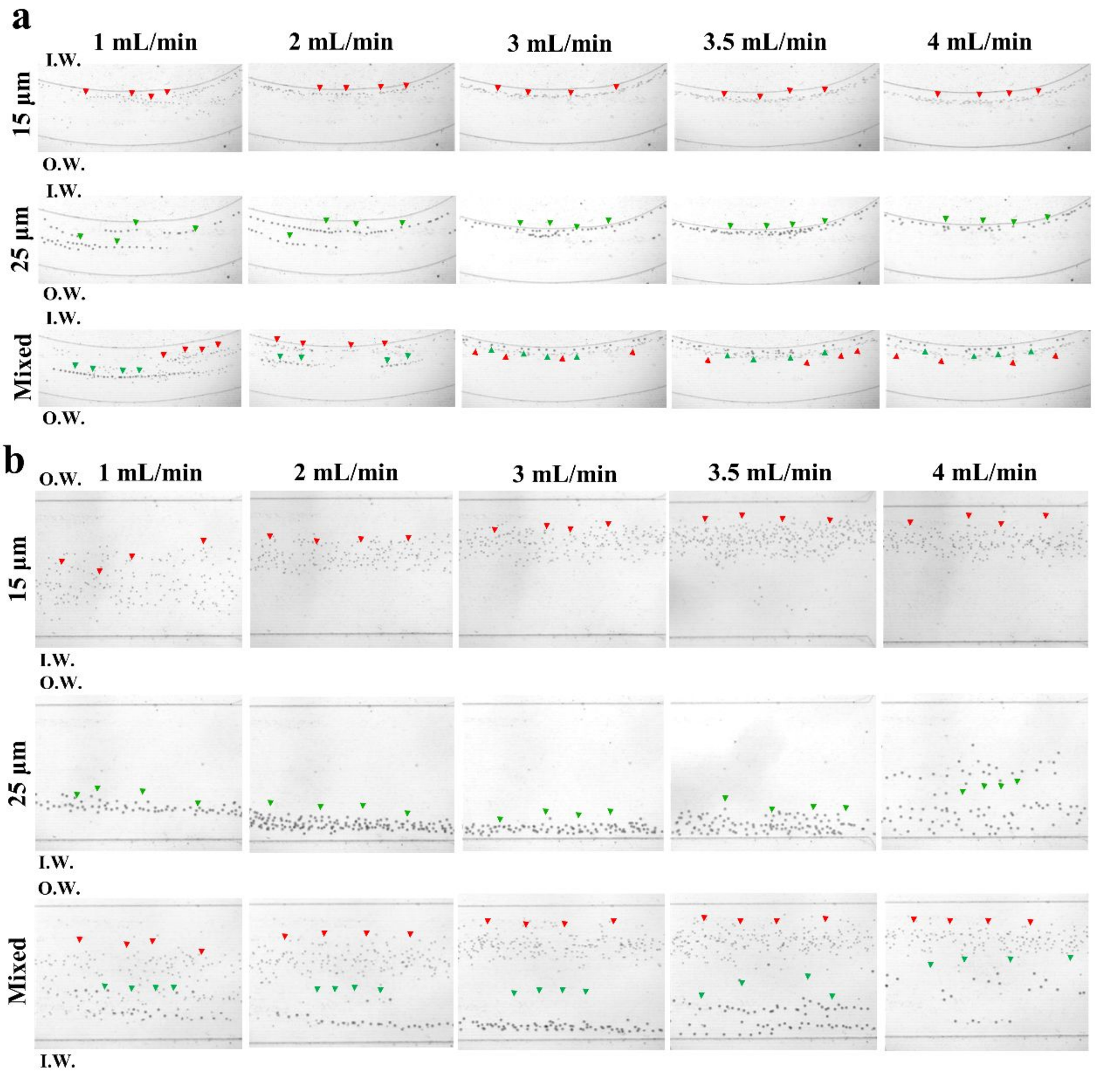

Figure S7. Testing of particle focusing within the MDDS device. High-speed camera images showing the lateral focusing of 15 μm (red arrows) and 25 μm (green arrows) polystyrene beads at the end of the 1st (a) and 2nd spiral (b) channels under the indicated flow rates. I.W.: inner wall. O.W.: outer wall.

References

(1) Yin, L.; Wu, Y.; Yang, Z.; Tee, C. A.; Denslin, V.; Lai, Z.; Lim, C. T.; Lee, E. H.; Han, J. Microfluidic label-free selection of mesenchymal stem cell subpopulation during culture expansion extends the

chondrogenic potential in vitro. *Lab on a chip* **2018**, *18* (6), 878-889. DOI: 10.1039/c7lc01005b From NLM.
(2) Di Carlo, D.; Irimia, D.; Tompkins, R. G.; Toner, M. Continuous inertial focusing, ordering, and separation of particles in microchannels. *Proceedings of the National Academy of Sciences of the United States of America* **2007**, *104* (48), 18892-18897. DOI: 10.1073/pnas.0704958104 From NLM.
(3) Bhagat, A. A.; Kuntaegowdanahalli, S. S.; Papautsky, I. Continuous particle separation in spiral microchannels using Dean flows and differential migration. *Lab on a chip* **2008**, *8* (11), 1906-1914. DOI: 10.1039/b807107a From NLM.
(4) Hou, H. W.; Warkiani, M. E.; Khoo, B. L.; Li, Z. R.; Soo, R. A.; Tan, D. S.; Lim, W. T.; Han, J.; Bhagat, A. A.; Lim, C. T. Isolation and retrieval of circulating tumor cells using centrifugal forces. *Sci Rep* **2013**, *3*, 1259. DOI: 10.1038/srep01259 From NLM.
(5) Wu, L.; Guan, G.; Hou, H. W.; Bhagat, A. A.; Han, J. Separation of leukocytes from blood using spiral channel with trapezoid cross-section. *Anal Chem* **2012**, *84* (21), 9324-9331. DOI: 10.1021/ac302085y From NLM.
(6) Guan, G.; Wu, L.; Bhagat, A. A.; Li, Z.; Chen, P. C.; Chao, S.; Ong, C. J.; Han, J. Spiral microchannel with rectangular and trapezoidal cross-sections for size based particle separation. *Sci Rep* **2013**, *3*, 1475. DOI: 10.1038/srep01475 From NLM.
(7) Jeon, H.; Jundi, B.; Choi, K.; Ryu, H.; Levy, B. D.; Lim, G.; Han, J. Fully-automated and field-deployable blood leukocyte separation platform using multi-dimensional double spiral (MDDS) inertial microfluidics. *Lab on a chip* **2020**, *20* (19), 3612-3624. DOI: 10.1039/d0lc00675k From NLM.
(8) Jeon, H.; Perez, C. R.; Kyung, T.; Birnbaum, M. E.; Han, J. Separation of Activated T Cells Using Multidimensional Double Spiral (MDDS) Inertial Microfluidics for High-Efficiency CAR T Cell Manufacturing. *Anal Chem* **2024**, *96* (26), 10780-10790. DOI: 10.1021/acs.analchem.4c01981 From NLM.
